# Psychobiotics improve propionic acid-induced neuroinflammation in juvenile rats, rodent model of autism

**DOI:** 10.1515/tnsci-2022-0226

**Published:** 2022-09-08

**Authors:** Mona Alonazi, Abir Ben Bacha, Anwar Al Suhaibani, Ahmad T. Almnaizel, Hisham S. Aloudah, Afaf El-Ansary

**Affiliations:** Biochemistry Department, Science College, King Saud University, Riyadh 11495, Saudi Arabia; Laboratory of Plant Biotechnology Applied to Crop Improvement, Faculty of Science of Sfax, University of Sfax, Sfax, Tunisia; Experimental Surgery and Animal Laboratory, College of Medicine, King Saud University, Riyadh, Saudi Arabia; Central Laboratory, King Saud University, Riyadh, Saudi Arabia; Biochemistry Department, Science College, King Saud University, P.O Box 22452, Riyadh 11495, Saudi Arabia

**Keywords:** bee pollen, probiotic, neurotoxic, autism, cytokines, psychobiotics

## Abstract

This study aimed to evaluate the protective and therapeutic potency of bee pollen and probiotic mixture on brain intoxication caused by propionic acid (PPA) in juvenile rats. Five groups of six animals each, were used: the control group only receiving phosphate-buffered saline; the bee pollen and probiotic-treated group receiving a combination of an equal quantity of bee pollen and probiotic (0.2 kg/kg body weight); the PPA group being treated for 3 days with an oral neurotoxic dose of PPA (0.25 kg/kg body weight); the protective and therapeutic groups receiving bee pollen and probiotic mixture treatment right before and after the neurotoxic dose of PPA, respectively. The levels of interleukin (IL)-1ß, IL-6, IL-8, IL-10, IL-12, tumor necrosis factor α, and interferon γ (IFN-γ) were investigated to evaluate the neuroinflammatory responses in brain tissues from different animal groups. The much higher IL-1β, IL-8, and IFN-γ, as pro-inflammatory cytokines (*P* < 0.001), together with much lower IL-10, as anti-inflammatory cytokine (*P* < 0.001) compared to controls clearly demonstrated the neurotoxic effects of PPA. Interestingly, the mixture of bee pollen and probiotics was effective in alleviating PPA neurotoxic effects in both therapeutic and protective groups demonstrating highly significant changes in IL-1β, IL-8, IL-10, and IFN-γ levels together with non-significant reduction in IL-6 levels compared to PPA-treated rats. Overall, our findings demonstrated a new approach to the beneficial use of psychobiotics presenting as bee pollen and probiotic combination in neuroinflammation through cytokine changes as a possible role of glial cells in gut–brain axis.

## 1 Introduction

Autism spectrum disorder (ASD) refers to an assembly of complex neurodevelopmental disorders showing impaired communication skills, deficits in social interaction, and restricted and stereotypic behaviors [1,2]. Mounting evidence from both clinical observations of ASD patients and rodent models clearly reveals that inflammatory responses strongly contribute to the pathophysiology of various neurodevelopmental diseases, particularly ASD. Propionic acid (PPA) is a short-chain fatty acid that is produced in the human body either as intermediary metabolite of fatty acid metabolism and a fermentation end product of certain enteric gut microbiota such as clostridia. Although largely derived in the gut, PPA and related short-chain fatty acids can readily cross the blood–brain barrier and get access to the brain, where they can induce numerous neurophysiological processes causing alteration of brain function and behavior [3].

Indeed, neuroinflammation is characterized by the activation of resident glial cells, committed to central nervous system (CNS) immune surveillance, by releasing chemokines, cytokines, and many other mediators, which, subsequently, recruit peripheral cells, including neutrophils, monocytes, and lymphocytes [4,5].

It is widely documented that immune responses could disturb both nervous and endocrine systems’ functions. The brain receives immune signals from the circulating cytokines (tumor necrosis factor [TNF]-α, interleukin [IL]-1β, IL-6, IL-8, IL-12, etc.) transported via the blood–brain barrier, from immune cells and from peripheral immunity through the vagus nerve, passing into the brain and its immune microglial cells [6,7].

Interestingly, IL-10, the powerful anti-inflammatory cytokine, displays a crucial role in balancing immune responses as a way to avoid chronic inflammatory diseases [8]. In fact, it acts in both adaptive and innate immunity, in respect of immunostimulatory and immunosuppressive effects, therefore, regulating response in several cell types, for instance antigen-presenting cells, such as macrophages, Langerhans cells, and dendritic cells [9].

As a way to ensure survival of the nervous tissue and alleviate inflammatory responses, the brain IL-10 levels remarkably rise during CNS pathology, hence, activating numerous signaling pleiotropic pathways [10].

Environmental factors including exposures to physical, chemical, psychological, microbial, and stressors strongly disrupt the functions of the immune-neuroendocrine network, defined as the communications between nervous, endocrine, and immune systems. Therefore, PPA could be considered as a stress agent for both nervous and immune systems. It might affect brain function by a number of ways causing the behavioral impairments previously reported [11]. Histological investigation of rodent’ brain tissue has reported the ability of PPA to activate microglia and reactive astrogliosis in the neocortical white matter and hippocampus, therefore, inducing an innate neuroinflammatory response [12,13]. It is well known that neuroinflammation occurs in many other diseases, particularly in Parkinson's and Alzheimer's disease, which suggest that this response could alter normal cognitive functioning [14,15]. Activated microglia secretes toxic substances such as nitric oxide and cytokines which are potentially damaging to neurons and may impair several functions of the brain [16].

A direct connection between the brain and gastrointestinal function has been reported in several studies. The “gut-microbiota-brain axis,” which is the ultimate link between the gut and the brain, is, indeed, a bidirectional communication system implicated in humoral and neuronal mechanisms [17]. Therefore, consumption of probiotics (live bacteria) aiming to balance the microbiota could be an interesting strategy to prevent or even to treat certain disorders. Nowadays, more and more researchers' attention has been attracted by the emerging evidence that some bacteria may exhibit positive mental health benefit (psychobiotic).

Bee pollen is an apitherapeutic product that is composed of amino acids, lipids, flavonoids, vitamins, and micronutrients. It demonstrates antifungal, antimicrobial, anti-inflammatory, and immunostimulating effects [18,19]. Bee pollen also exhibits anti-inflammatory mechanisms through the inhibition of the activities of cyclooxygenase and lipoxygenase, the enzymes that are responsible for the conversion of arachidonic acid into toxic compounds as prostaglandin and leukotrienes as inducers of acute and chronic inflammatory conditions in different tissues [18,19].

Probiotics and prebiotics show several potential protective and therapeutic effects against numerous human diseases such as gut disorders, colorectal cancer, inflammatory bowel disease, autism, carcinogenesis, etc. However, little scientific research explored the beneficial effects of prebiotics and probiotics on neuroinflammation. This study as complementary study to our most recent published works [20,21,22,23,24] aims to evaluate the ameliorating effects of symbiotics as combination of probiotics and prebiotics on PPA-induced neuroinflammation as etiological mechanism in ASD through the measurement of interferon gamma (IFN)-α, TNF-α and IL-1β, 6, 8, 10, and 12 in the brain of juvenile rats.

## 2 Materials and methods

### 2.1 Animal experiments

**Figure 1:**
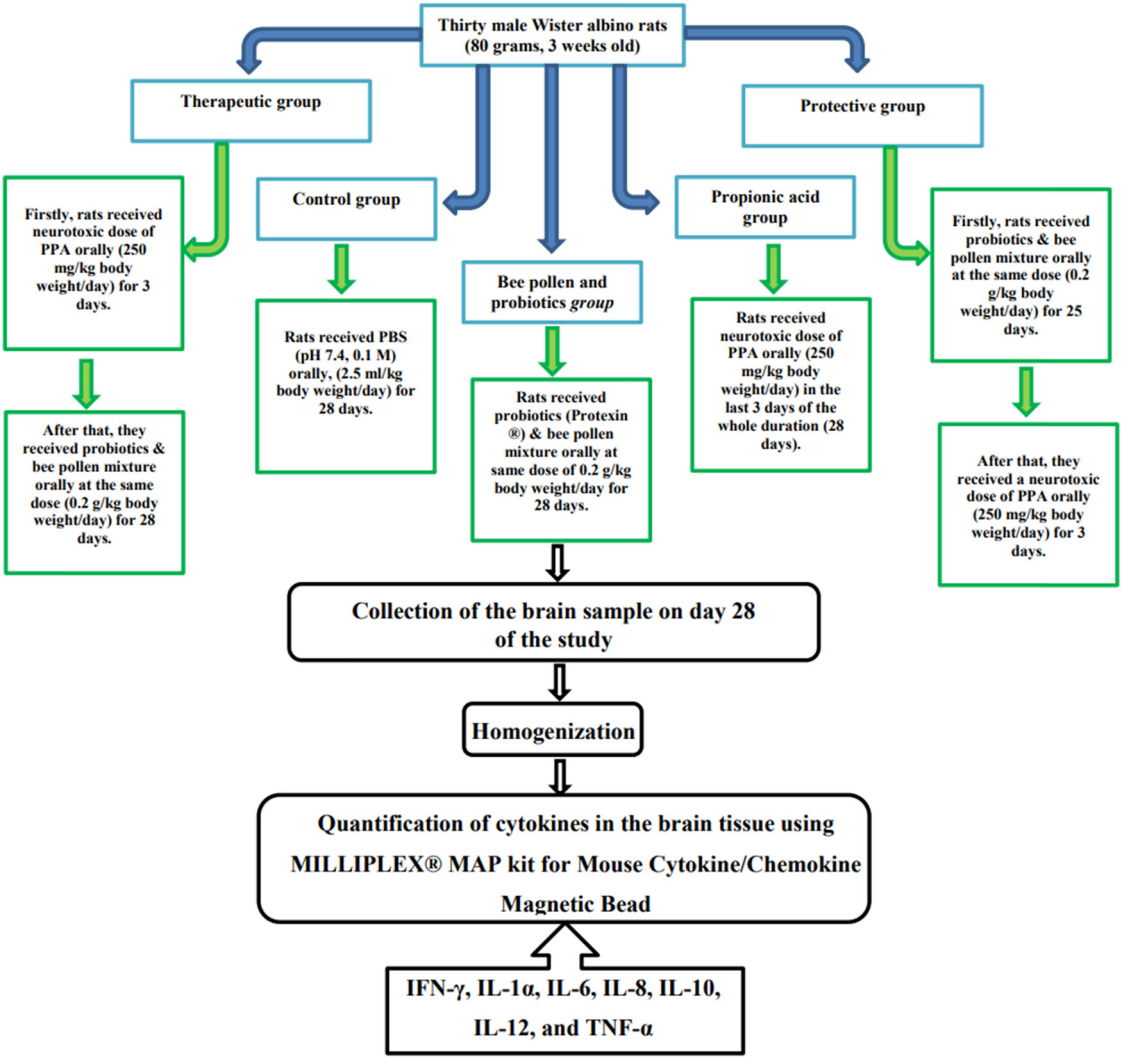
Diagrammatic scheme of the animal experiments. PPA was a product of Sigma-Aldrich (USA). Bee pollen (NZ Bee Pollen Granules) and probiotics (PROTEXIN^®^) were products of Happy Valley (New Zealand) and Probiotics International Limited (UK), respectively.

The present study’s experiments were carried out on 30 Wistar albino rats, weighing 80 g and 3 weeks old. They were housed in groups in cage (26.5 cm × 14.5 cm × 42.5 cm) under controlled laboratory conditions (temperature 23°C, humidity 55 ± 5% and day/night 12 h light cycle). All animals had free access to standard diet (AIN-93 G) obtained from Grain Silos and Flour Mills Organization, Riyadh, Saudi Arabia. The experimental procedure was pre-approved by the ethics committee for animal research of King Saud University, Riyadh (ethics reference number: KSU-SE-19-35). Rats were placed on cages, 3 rats in each for 1 week to become acclimatized, then all rats were randomly divided into 5 groups, with 6 rats in each group as shown below (Figure 1): *Control group* received phosphate buffered saline; *Bee pollen and probiotics-treated group* was orally administered with a combination of an equal quantity of bee pollen and probiotic at a dose of 200 mg/kg body weight; *PPA-treated group* received a dose of PPA of 250 mg/kg body weight consecutively for 3 days; *Therapeutic group* was administered with PPA followed by bee pollen and probiotics combination at the dose 200 mg/kg body weight; *Protective group* received bee pollen and probiotics combination orally followed by PPA. The bee pollen granules were first weighed, grinded, and then dissolved in distilled water with probiotics and orally administered to animals with oral gavage.

On day 28, rats had been euthanized using CO_2_, and their brains were dissected carefully from the skull. One gram of each brain tissue was homogenized in 10 mL of 10% phosphate buffered saline for 5–10 s, then, centrifuged at 3,000 rpm at 4°C for 10 min. The resultant supernatants were stored at –80°C until use.


**Ethical approval:** The research related to animals’ use has been complied with all the relevant national regulations and institutional policies for the care and use of animals. The experimental procedure was pre-approved by the ethics committee for animal research of King Saud University, Riyadh (ethics reference number: KSU-SE-19-35).

### 2.2 Quantification of cytokines in the brain tissue

MILLIPLEX^®^ MAP kit for Mouse Cytokine/Chemokine Magnetic Bead was used to measure a panel of cytokines including: IFN-γ, IL-1ß, IL-6, IL-8, IL-10, IL-12, and TNF-α following the manufacturer’s instructions.

### 2.3 Statistical analysis

All data were carried out by *one-way ANOVA* followed by Tukey's Multiple Comparison Test. *P* values ≤0.05 were considered significant. Results were illustrated as mean value ± standard deviation, using the statistical package for the social sciences (SPSS V21).

## 3 Results

**Figure 2:**
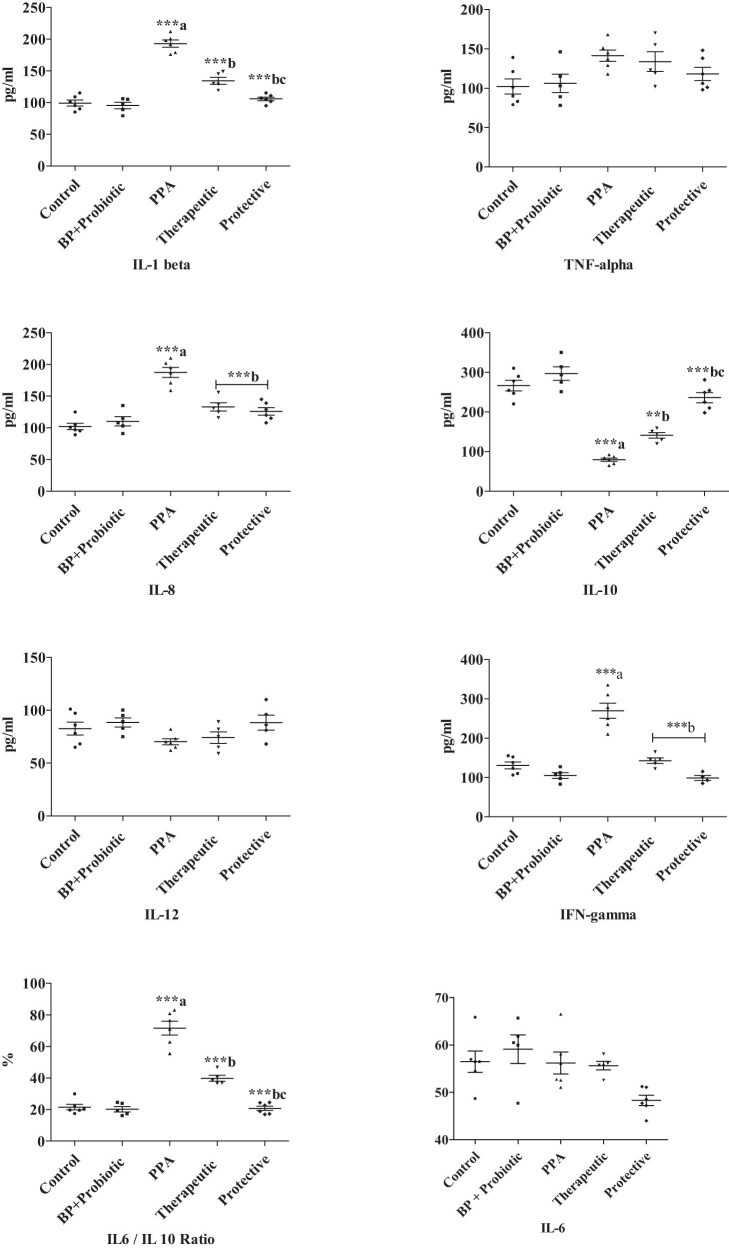
Mean value ± S.D. of all the measured parameters in brain homogenate of treated rats’ pups compared to control group. Only *P* values ≤0.05 were considered significant. ^a^ is the comparison between control vs PPA-treated groups; ^b^ is the comparison between PPA-treated group vs therapeutic and protective groups; ^c^ is the comparison between therapeutic vs protective groups.

Significantly higher levels of IL-1β (94.94% increase), IL-8 (83.62% increase), IFN-γ (106.35% increase), and TNF-α (38.25% increase) together with decreased IL-10 (66.97% decrease) and IL-12 (14.94%) levels were recorded in PPA-treated group compared to the untreated animals as described in Table 1 and Figure 2. The much higher IL-1β, IL-8, and IFN-γ, as pro-inflammatory cytokines (*P* < 0.001), together with much lower IL-10, as anti-inflammatory cytokine (*P* < 0.001) compared to controls clearly showed the neurotoxic effects of the PPA. Combined probiotics and bee pollen were effective in alleviating the PPA neurotoxic effects in both therapeutic and protective groups demonstrating highly significant changes in IL-1β, IL-8, IL-10, and IFN-γ together with non-significant reduced IL-6 levels compared to PPA-treated rats (Table 1 and Figure 2).

**Table 1:** Mean value ± S.D. of all the measured parameters in brain homogenate of treated rats’ pups compared to control group

Parameter	Control	BP + Probiotic	PPA	Therapeutic	Protective
IL-1β (pg/mL)	99.17 ± 11.43	95.4 ± 11.41	193 ± 13.68^***a^	134.4 ± 12.36^***b^	105.8 ± 6.998^***bc^
TNF-α (pg/mL)	102.2 ± 23.52	106.2 ± 26.28	141.3 ± 17.51	133.8 ± 27.87	118.2 ± 20.65
IL-8 (pg/mL)	102.0 ± 12.51	110.4 ± 16.30	187.3 ± 19.27^***a^	133.0 ± 15.02^***b^	126.0 ± 14.28^***b^
IL-10 (pg/mL)	266.5 ± 32.48	296.8 ± 37.77	79.50 ± 10.33^***a^	141.0 ± 15.83^***b^	236.2 ± 30.85^***bc^
IL-12 (pg/mL)	82.50 ± 14.84	88.40 ± 9.839	70.17 ± 6.969	74.00 ± 12.21	88.20 ± 15.82
IL-6 (pg/mL)	56.49 ± 5.52	59.14 ± 6.77	56.2 ± 5.69	55.65 ± 1.99	48.31 ± 2.7
IFN-ι (pg/mL)	130.7 ± 20.95	105.2 ± 16.47	269.7 ± 47.15^***a^	142.6 ± 15.82^***b^	98.75 ± 12.87^***b^
IL-6/IL-10 ratio	21.57 ± 4.364	20.23 ± 3.832	71.63 ± 10.75^***a^	39.81 ± 4.183^***b^	20.82 ± 3.477^***bc^

*P*-value <0.001 ***. ^a^is the comparison between control vs PPA – treated groups; ^b^is the comparison between PPA – treated group vs therapeutic and protective groups; ^c^is the comparison between therapeutic vs protective groups. BP, bee pollen; IL-, Interleukin; IFN-ι, interferon γ; TNF-α, Tumor necrosis factor α.

## 4 Discussion

The CNS and the immune system were considered, for a long time, as systems that operate independently and separately. Nevertheless, recent studies show an active communication between these two compartments, whereby they modulate each other “bi-directly” with neuromodulators and neurotransmitters in the periphery. Besides, the discovery of lymphatic vessels in the CNS put in check the current view of the brain as an “immune privileged site” and suggests novel potentials for the crosstalk between the brain and the immune system [25]. In the current study, in spite of the fact that oral gavage to the rats could possibly be associated with induced stress and immune suppression [26,27], it would not affect the tested ameliorative potential of psychobiotics as intervention in PPA- rodent model of ASD. It is documented that there is no test-substance-related evidence of immunosuppression in orally administered animals [28]. Based on the comparative nature of our study, where all the tested groups experienced oral gavage-induced stress for the same durations (28 days), either when orally received PBS in control, PPA in ASD model, PPA followed by psychobiotics or psychobiotics followed by PPA-treated groups, the oral administration would not affect the investigated potency of the intervention through the measurement of cytokines as biomarkers of inflammatory responses.

Once activated, microglial cells may commit to different “reactive” phenotypes which demonstrate a huge molecular and functional diversity. These variations in microglia profile are associated with the type of challenge faced by the CNS. They might move to a proinflammatory state, named the “M1 phenotype” [29], showing potent neurotoxic and phagocytic activities and liberating proinflammatory cytokines and chemokines in response to an immune challenge, such as the presence of proinflammatory signals [30,31,32] or a microorganism invasion [33].

The inflammatory role of cytokines has been involved in numerous neuropsychiatric pathologies, including autism. Alterations in the IL-1β system with abnormally increased serum levels in children with ASD were previously reported by ref. [34]. This could support the marked elevation of IL-1β in PPA-treated rodent model of ASD reported in this study (Table 1 and Figure 2). Our data also demonstrate the ameliorating effects of bee pollen and probiotics either as therapeutic or protective intervention. It is clear that the combined bee pollen and probiotic treatment as positive control group is safe enough and did not induce any changes in the measured cytokine profile. This collaborates with the recent findings of ref. [35] in which the potency of bee pollen as an effective natural compound in alleviating neuroinflammation and oxidative damage caused by immobilization stress in rat brain tissue was proved. The reported therapeutic and protective effects of probiotic is in good agreement with ref. [36] in which the efficacy of probiotics in the control of neurological disorders through the gut–brain axis was proved.

More or less, the same trend was observed regarding the neuroinflammatory effect of PPA or the therapeutic and protective effects of combined bee pollen and probiotics on the other set of measured cytokines (IFN-γ, IL-6, IL-8, IL-10, IL-12, and TNF-α). The Significant increase in IL-6 and TNF-α (Table 1 and Figure 2) can find support in numerous studies which demonstrate that intracerebroventricular or oral delivery of PPA in rats resulting in increased IL-6, TNF-α, IL‐1β, IL-1, and INF-γ cytokine levels in response to microglia over-proliferation. and up‐regulation of selective cytokines gene expression [12,13,37,38,39,40].

Moreover, it is in good agreement with ref. [41] which reported high levels of TNF-α, IL-1α, and IL-6 together with significant low IL-10 level in PPA-treated hamsters as indication of PPA neuroinflammatory effects (*P* < 0.05). Several studies have reported a connection between the IL-6 function and the acute phase inflammatory C-reactive protein (CRP). In fact, the pro-inflammatory cytokines, such as TNF-α and IL-1, stimulate CRP expression during IL-6 elevation.

Among the most common probiotics, *Bifidobacteria* and *Lactobacilli* exhibit considerable health promoting potencies including modifying the population and composition of gut microbiome and ameliorating the function of the intestinal barrier [42,43,44]. Furthermore, these probiotics facilitate the generation of metabolic intermediates like short-chain fatty acids [45,46] and diminish gut permeability [47], therefore, improving immune responses and reducing inflammation [42,46,48].

The reported anti-inflammatory effect of bee pollen could be attributed to its high content of flavonoids, as major antioxidant and anti-inflammatory ingredient. Flavonoids are known to inhibit the proinflammatory signaling pathways such as nitric oxide excessive release, and COX-2 over-expression through the prevention of NF-kB activation [49,50,51]. This could be supported by the fact that NF-kB, COX-2, and NO activation are all reported as etiological mechanisms associated with ASD [52,53,54].

The reported ameliorative effects of bee pollen as component of the used symbiotic (Table 1 and Figure 2) can find support in ref. [55] which proved that some flavonoids in bee pollen efficiently reduce the risk of inflammation related diseases. Flavonoids, for instance, kaempferol and quercetin that are the main flavanols of bee pollen [56], have been documented as possessing anti-inflammatory [55,57] and antioxidant [58] properties. Quercetin inhibits inflammatory cytokines for instance IL-8 implicated in the pathogenesis along with the activity of arachidonic acid metabolizing enzymes such as lipoxygenase, cyclooxygenase, and phospholipase A2.

According to ref. [59], psychobiotics are beneficial bacteria (probiotics) or support such bacteria (prebiotics) that affect bacteria–brain relationships. Numerous bacterial strains particularly *Bacillus breve*, *Bacillus animalis* subsp. *lactis*, *Lactobacillus helveticus*, *Lactobacillus acidophilus*, *Lactobacillus plantarum*, *Lactobacillus paracasei,* and *Streptococcus thermophilus* exhibit psychobiotic properties, [59,60,61]. During the last two decades, positive changes in neural activity in specific brain compartments implicated in cognition, mood, and emotional processing have been associated with probiotics utilization [62,63]. Moreover, specific probiotics have been found to reduce the levels of pro-inflammatory cytokines including TNF-α, IL-1, or IL-6 [64,65] often associated with certain psychiatric disorders [66,67]. Thus, these accumulating evidences strongly suggest that the immune-modulatory role of probiotics may be critical to counteract and/or improve inflammation-related brain deficits. Notably, mice treated with a single, peripheral dose of lipopolysaccharide from Gram (−) bacteria, show evident signs of neuro-inflammation and “sickness behavior” [68,69].

In contrast, IL-10 was significantly lower in PPA-rodent model of autism, and much higher in bee pollen and probiotics treated and protected rats which show anti-inflammatory effects, shown as marked reductions in IL-1ß, IL-8, TNF-α, and IFN-γ, and a significant elevation of the anti-inflammatory cytokine IL-10. These findings collaborate with those previously reported in ref. [70], which demonstrates that propolis and bee pollen contain bioactive compounds in the floral origin of honey bee and plants. These biomolecules may act upon both adaptive and innate immune responses. These active substances significantly reduce superoxide anion production and pro-inflammatory cytokine synthesis in rabbit neutrophils. Compared to propolis, bee pollen shows relatively lower activity, particularly in terms of the induction of IL-10. This could be attributed to the higher level of the anti-inflammatory and anti-oxidant compounds, and polyphenols in propolis than in bee pollen [71].

This could be explained in the basis that some probiotics among which is *Bifidobacterium* spp. are representative probiotics that play an important role in their hosts’ health. They display relatively elevated cell adhesion to colonic cells in addition to their numerous *in vivo* and *in vitro* bio functionalities, particularly, modulatory effects on immune cells [72].

Bee pollen and probiotics seem to be effective in improving PPA neuroinflammatory effects and this can be observed as much lower IL-6/IL-10 recording values of 39.81 ± 4.183 and 20.82 ± 3.477 in the treated and protected groups, respectively, compared to a remarkably higher ratio in PPA-treated rats showing a value of 71.63 ± 10.75. This could be supported by the recent work of our research team in which treatment using *Bifidobacterium* was remarkably effective in alleviating the altered gut microbiota as an autistic feature in orally administering PPA-rodent model of autism [11]. Moreover, it can demonstrate the synergistic effects of combining prebiotic (bee pollen) and probiotic (mixture of beneficial bacteria) as anti-inflammatory treatment strategy of PPA-induced neuroinflammation [73,74].

In conclusion, our findings demonstrated a new approach to the beneficial use of psychobiotics presenting as bee pollen and probiotic combination in neuroinflammation through cytokine changes as a possible role of glial cells in gut–brain axis.
